# Food-Intolerance Genetic Testing: A Useful Tool for the Dietary Management of Chronic Gastrointestinal Disorders

**DOI:** 10.3390/nu16162741

**Published:** 2024-08-16

**Authors:** Alexandra Celi, María Trelis, Lorena Ponce, Vicente Ortiz, Vicente Garrigues, José M. Soriano, Juan F. Merino-Torres

**Affiliations:** 1 Joint Research Unit on Endocrinology, Nutrition and Clinical Dietetics, Health Research Institute La Fe, 46026 Valencia, Spain; micecal@alumni.uv.es (A.C.); jose.soriano@uv.es (J.M.S.); merino_jfr@gva.es (J.F.M.-T.); 2 Parasite & Health Research Group, Area of Parasitology, Department of Pharmacy and Pharmaceutical Technology and Parasitology, University of Valencia, 46010 Valencia, Spain; 3 Department of Bioinformatics, Overgenes S.L., 46980 Valencia, Spain; lorena.ponce@overgenes.com; 4 Digestive Functional Disorders Unit, Department of Gastroenterology, University and Polytechnic Hospital La Fe, 46026 Valencia, Spain; ortiz_vicbel@gva.es (V.O.); garrigues_vic@gva.es (V.G.); 5 Department of Medicine, Faculty of Medicine, University of Valencia, 46010 Valencia, Spain; 6 Food & Health Lab, Institute of Materials Science, University of Valencia, 46980 Valencia, Spain; 7 Department of Endocrinology and Nutrition, University and Polytechnic Hospital La Fe, 46026 Valencia, Spain

**Keywords:** carbohydrate intolerance, celiac disease, genetic testing, malabsorption, lactose, fructose

## Abstract

The rise in food intolerances and celiac disease, along with advanced diagnostic techniques, has prompted health professionals to seek effective and economical testing methods. This study evaluates combining genetic tests with routine carbohydrate-absorption breath tests to classify patients with chronic gastrointestinal disorders into therapeutic groups, enhancing dietary management and improving gut health and quality of life. Forty-nine patients with suspected carbohydrate intolerance underwent genetic testing for lactase non-persistence, hereditary fructose intolerance, and celiac disease risk. Simultaneously, breath tests assessed lactose and fructose absorption. The lactase non-persistence genotype appeared in 36.7% of cases, with one hereditary fructose-intolerance case in a heterozygous condition. Celiac disease risk markers (HLA-DQ2/8 haplotypes) were found in 49.0% of the population. Secondary lactose and/or fructose malabsorption was present in 67.3% of patients, with 66.1% of lactase non-persistence individuals showing secondary lactose malabsorption. Fructose malabsorption was prevalent in 45.8% of patients at risk for celiac disease. Two main treatment groups were defined based on genetic results, indicating primary and irreversible gastrointestinal disorder causes, followed by a sub-classification using breath test results. Genetic testing is a valuable tool for designing dietary management plans, avoiding unnecessary diet restrictions, and reducing recovery times.

## 1. Introduction

In recent years, the prevalence of food allergies (FAs) and food intolerances (FIs) has significantly increased in humans. This is likely to be due to changes in dietary habits, particularly in developed countries, leading to imbalanced diets that also promote overweight and obesity [1]. Furthermore, there is now a greater awareness among the population regarding the impact of diet on health, and there is also a wider, more easily accessible, range of diagnostic tests for food allergies and intolerances [2]. However, several of these tests lack scientific validity and can lead to incorrect diagnoses and unnecessary dietary restrictions, affecting both intestinal health and quality of life [2,3]. This wide range of tests has prompted health professionals to question which ones to apply in each case, and at the public health level, which are best in terms of cost-effectiveness. It has been estimated that 15% to 20% of the population suffer from food intolerances [3,4], and food allergies affect 3% to 10% of adults and 8% of children worldwide [5]. However, it is known that both the prevalence of food allergies and intolerances identified using more rigorous diagnostic methods is significantly less common than those perceived or self-reported by patients. Additionally, the definitions of “intolerance”, “allergy” and “sensitivity” to certain foods, among other terms, are used interchangeably and almost randomly without truly understanding what each pathology implies, thus contributing to confusion and inaccurate prevalence percentages [3,5,6,7].

Both FAs and FIs are adverse reactions to food, but the fundamental difference is that the former is immunologically mediated, while the latter is not [1,6,7]. Therefore, although these terms can easily be confused due to a lack of knowledge, their pathological mechanisms, diagnosis, and treatment are different [7], and an inadequate approach can lead to severe nutritional deficiencies and other adverse effects on patients’ health and quality of life [1,7]. FI is initiated by a food or food component at a normally tolerated dose, often related to the malabsorption of certain sugars, fats, proteins or vitamins as they pass through the small intestine [2]. Hence, the presence of lactose malabsorption, for example, is necessary, but not sufficient, to diagnose lactose intolerance, as the presence of symptoms is essential for the diagnosis of intolerance [7]. Intolerances, unlike allergies, are generally characterized by being dose-dependent and related to intestinal malabsorption processes [1,7]. For example, a person with lactose intolerance will not manifest the same symptom profile after ingesting 40 g of aged cheese, which contains less lactose than fresh cheese, as after drinking a glass of milk [1].

The type of clinical manifestation is associated with the causal mechanism of intolerance [1]. This work mainly focuses on intolerances of which the origin is related to malabsorption, specifically lactose and fructose. The accumulation of these substrates causes an osmotic effect due to the accumulation of fluid secretion in the intestinal lumen, usually causing diarrhea and other symptoms such as flatulence, alteration of bowel movements, abdominal distension, and pain due to the fermentation of non-absorbed sugars by the bacteria in the colon [1].

Some causes of malabsorption are directly related to genetic markers of hereditary intestinal alterations, such as the non-persistent lactase genotypes in primary lactose, but also indirect genetic predisposition related to the HLA-DQ2/8 haplotypes in celiac disease [1,8,9]. Additionally, there are several inflammatory conditions that could alter the integrity of the mucosa and therefore the expression of enzymes such as lactase or fructose transporters, including small intestinal bacterial overgrowth (SIBO), inflammatory bowel disease (IBD), dietary components, small intestine infections (viruses, bacteria, and parasites), drugs (nonsteroidal anti-inflammatory), etc. [1,10,11]. Previous studies of our group led to the routinary recommendation of breath tests (lactose and fructose). In addition, the recommended algorithm for the diagnosis and dietary–nutritional management of patients with chronic gastrointestinal disorders (CGDs) suggested the inclusion of genetic tests for primary lactose intolerance, hereditary fructose intolerance and celiac disease risk [12].

For effective management, it is important to determine which tests or combination of tests are appropriate for classifying patients into different treatment groups, with the aim of obtaining more successful outcomes with dietary management and avoiding subjecting patients to an unnecessarily long and exhausting diagnostic process [7].

## 2. Materials and Methods

This is a cross-sectional study on the applicability of the combination of a genetic approach to hereditary intestinal alterations and traditional carbohydrate breath tests to guide the management of patients with chronic gastrointestinal disorders (CGDs) and achieve the greatest benefits for them.

### 2.1. Ethical Approval

Written consent was obtained from all participants after they were informed about the aim of the study, including risks and implications of their participation in it, as well as the treatment and confidentiality of the data. This study was approved by the Biomedical Research Ethics Committee of University and Polytechnic Hospital La Fe (Project identification code: 2019/0100), respecting the fundamental principles of the Declaration of Helsinki and the Council of Europe Convention in relation to Human Rights and Biomedicine of the UNESCO Declaration. Likewise, each participant signed a specific consent form for the genetic analysis conducted by the company Overgenes, S.L. (Valencia, Spain).

### 2.2. Patients’ Description and Clinical Profiles

Forty-nine patients (♀, *n* = 31; ♂, *n* = 18) aged 17 to 70 referred to the Gastroenterology Department at La Fe University Hospital with suspected carbohydrate intolerance and/or malabsorption and the presence of at least two of the main digestive symptoms associated with CGD—such as abdominal distension or pain, bloating, borborygmi and altered bowel habits, among others—for at least 3 months were included in the study. They were recruited from November 2022 to June 2023. Exclusion criteria included antibiotic or anthelmintic use in the last 30 days, gastrointestinal bleeding, neoplastic history of the gastrointestinal tract, chronic treatments with nonsteroidal anti-inflammatory drugs and previous abdominal surgery.

Patients were interviewed to gather relevant information through a standardized questionnaire that included personal and family history and the presence of digestive and non-digestive symptoms.

### 2.3. Genetic Determinations

A blood sample was taken to simultaneously analyze the presence or absence of the genetic predisposition to non-persistent lactase, hereditary fructose intolerance and celiac disease, by the “Myi3 Food Intolerance Test” (Overgenes, S.L.). Specifically, this genetic test analyzes the presence, in homozygosity or heterozygosity, of the following genetic markers: (a) primary lactose intolerance—five single nucleotide polymorphisms (SNPs) in the LTC/MCM6 (lactase promoter) gene; (b) hereditary fructose intolerance—seven mutations in the Aldolase B (ALDOB) gene; and (c) celiac disease risk—human leukocyte antigen (HLA) genetic system.

The methodology applied to perform the DNA test was a high-capacity DNA amplification and sequencing system such as massive or next generation sequencing (NGS). The NGS methodology enables the parallel and precise generation of millions of DNA fragments in a single, rapid sequencing process. This methodology allows for amplifying and sequencing each of the analysis regions at least 100 times, thus enabling the identification of each analyzed genotype with an accuracy of 99.99%. The NGS platform used for the test was Illumina’s MiSeq (MiSeq™ System, San Diego, CA, USA).

### 2.4. Breath Tests

During the first interview, three specific kits—those for small intestinal bacterial overgrowth (SIBO), lactose malabsorption, and fructose malabsorption—were provided to each patient: SIBOkit, Lactokit and Fructokit (Isomedpharma, Madrid, Spain), respectively. The glucose breath test (GBT) for the determination of SIBO was carried out in parallel to the carbohydrate test, as a positive result would render the results of the other two tests useless by demonstrating an intestinal bacterial overgrowth that would per se affect gas production.

According to the hospital protocol, the tests were taken at home and then returned for analysis. The tests were performed at least every 24 h, but in case of discomfort or digestive symptoms different from usual, patients were advised to wait 48 to 72 h before performing the next test. The breath samples were collected in a series of test tubes, and alveolar gas samples were analyzed for hydrogen (H_2_) and methane (CH_4_) using gas chromatography. The values of the three tests were proportionally corrected to a value of 5.5% carbon dioxide (CO_2_) according to the specifications of the analyzer.

Prior to conducting the breath tests, according to the hospital protocol, patients were asked to suspend the use of probiotics for at least two weeks and the use of laxatives and enemas for 3 days, and not to have undergone colonoscopy in the last 30 days. In addition, the night before the test, they were asked to follow a low-residue carbohydrate diet starting at dinner, to not engage in physical activity after dinner, and to remain fasting for at least 10 h before the test.

For the lactose and fructose breath test (LBT and FBT), patients received 25 g of lactose or fructose, respectively, to be diluted in 250 mL of water. One base sample and seven post-lactose-/-fructose-ingestion samples were requested to be taken every 25 min. According to the “Isomed Diagnostics Laboratory”, carbohydrate malabsorption is determined when there is an elevation after 90 min of H_2_ concentration greater than 20 ppm and/or an elevation of CH_4_ greater than 12 ppm with respect to the baseline value. When the increment of gases is accompanied by symptoms associated with malabsorption, it is defined as intolerance. For the SIBO breath test, patients received 75 g of glucose to be diluted in 250 mL of water, or in cases of patients with diabetes, lactitol was used instead of glucose. One base sample and eleven post-glucose-ingestion samples were requested to be taken every 15 min. The reference values for determining SIBO are a concentration of H_2_ greater than 15 ppm and/or an elevation of CH_4_ greater than 10 ppm, with respect to the baseline value before 90 min.

### 2.5. Statistical Analyses

For the analysis of categorical variables (genetic markers, presence of malabsorption and symptoms), absolute and relative frequencies were calculated. In addition, when the sample size was sufficient, comparisons of categorical variables were performed with Pearson’s chi-squared test or Fisher’s exact test, as appropriate. A XP-value below 0.05 (typically  ≤  0.05) was considered statistically significant. Data were analyzed using the statistical software R (4.3.0 version).

## 3. Results

### 3.1. Characteristics of the Patients Enrolled in the Study at Baseline

The sociodemographic and baseline clinical characteristics of the patients are summarized in Table 1. The four main gastrointestinal symptoms presented by the patients, affecting more than half of the population, were altered gut transit (83.7%), abdominal distension (79.6%), followed by flatulence (69.4%) and abdominal pain (69.4%). Altered gut transit, either diarrhea (34.7%), constipation (20.4%) or both (28.6%), was present in one-third of the cases. Regarding the extraintestinal symptoms, the most frequent were skin itching (20.4%), articular pain (16.3%) and fatigue (16.3%).

### 3.2. Genetic Testing Results

At the genetic level, there are currently five known single nucleotide genetic polymorphisms (SNPs) associated with the lactase persistence phenotype; two of them are more frequent in populations of Caucasian origin (C/T-13910 and G/A-22018), and the other three are more frequent in populations of African origin (C/T-14010, T/G-13915 and C/G-13907). Genetic results for primary lactose intolerance, related to a lactase non-persistence (LNP) condition, are summarized in Table 2. The genetic differences appeared in the Caucasian SNPs, with 36.7% of the patients presenting the homozygous risk variant for an LNP phenotype (Table 2). Only one case of hereditary fructose genetics was detected, namely a heterozygous carrier of the ALDOB mutation, considered to have normal fructose metabolism. The prevalence of genetic predisposition for celiac disease (CD) was present in almost half of the cases (49.0%). Regarding the HLA-DQ2/8 haplotypes marker, the patients were classified into different risk levels based on the genetic variants identified [13] (Table 3). There is a small group of six patients with the highest CD genetic risk, which is determined by the homozygous DQ2.5 haplotype or the DQ2.5/DQ2.2 genotype (12.2%). However, the moderate level was the most frequent in this group of symptomatic patients (32.7%), with a clear predominance of the genotype DQ2half in 12 cases (24.5%). Finally, two of the at-risk patients had a genotype with a low predisposition to develop the disease with a DQ2.2/DQ-genotype (4.1%) (Table 3).

### 3.3. Genetic Results and Carbohydrate Malabsorption by Breath Test

Twenty-three patients (67.3%) were diagnosed with secondary lactose and/or fructose malabsorption. The coexistence of genetic risk for hereditary intestinal disorders and carbohydrate malabsorption, determined by breath test, was summarized in Table 4. Among individuals with the LNP phenotype, the most remarkable, but expected finding is a high frequency of lactose malabsorption (66.1%) and five cases (27.8%) of fructose malabsorption. It is also worth mentioning that one-third of these patients presented SIBO (33.3%), and their carbohydrate absorption could therefore not be assessed. Within the group of patients at risk for CD, the prevalence of fructose malabsorption reached the highest values (45.8%). One-third had lactose malabsorption and five individuals had combined malabsorption of both carbohydrates. Concerning the results in this group of patients, it should be noted that SIBO was diagnosed on seven occasions; thus, no data for the lactose and fructose tests were available in those cases.

Finally, in positive cases of celiac disease genetics, a sub-analysis of the absorption capacity of carbohydrates according to the classification of the risk level was performed (Table 5). Among individuals at high risk of CD, a malabsorption of both lactose and fructose was detected in 33.3% of individuals, taking into account that 50% of the members of the group could not be assessed in this sense as they tested positive for SIBO. Regarding cases of moderate risk (the largest group of patients), 31.3% of lactose malabsorption was obtained, and 50% of the evaluated individuals presented fructose malabsorption. SIBO in this moderate group turned out to be less frequent than in the previous one.

### 3.4. Treatment Groups Based on Genetic Testing and Breath Test Results

Genetic risk and breath test assessments were used to classify patients into therapeutical groups. Two main groups of treatment were initially defined based on genetic characteristics as they are possible primary and irreversible causes of gastrointestinal disorders: (a) lactase non-persistence (LNP) condition and (b) HLA of risk or genetic predisposition to CD. SIBO and negative-breath-test patients will not be addressed in depth in this article as it is focused on the patients who present malabsorption. Patients with SIBO might require a different treatment approach, considering this overgrowth situation, and are therefore addressed as a separate group.

For genetic predisposition for LNP, a first subgroup within this condition would be the patients that combine genetic intolerance with secondary lactose malabsorption (LM), classified as LNP/LM—a condition that 10 of the 18 with LNP meet. These patients will be given specific nutritional recommendations, mainly focused on low lactose content in their diet. Our findings, in accordance with previous studies, confirm that there is a correlation between the positive genetics for LNP and the condition of secondary lactose malabsorption (*p* = 0.0003268). A second subgroup would present fructose malabsorption (FM) alone or in combination with LNP genetics (LNP/FM). Besides the lactose restriction, this group needs to take into account their fructose malabsorption in their dietary management. Furthermore, causes other than LNP should be considered as primary causes of malabsorption, such as SIBO, chronic intestinal infection, or positive HLA-DQ2/8, which will be discussed later.

For genetic predisposition to celiac disease, in the genetic situation of HLA with a risk of celiac disease, a sub-classification of patients according to the breath test results was established. The first subgroup would include subjects with the HLA/LM condition and without LNP. In these cases, if there is no positive response to a low-lactose diet, alternative diet approaches, such as a gluten-free diet, will have to be tried. A second treatment group would be constituted by patients who combine HLA and fructose malabsorption (HLA/FM)—a combination that has turned out to be highly prevalent in our study (45.8%), which will initially require a diet with restricted fructose content for symptom control. If there is a no positive response to the low-fructose diet, primary causes of malabsorption need to be assessed, and a gluten-free diet should be considered.

## 4. Discussion

Chronic gastrointestinal disorders have become an increasingly common health problems, representing a burden to patients by the symptoms generated, the reduced quality of life, and the costs associated with them [14]. The prevalence varies across different countries, but the general trend is a higher incidence in high developed countries. Primarily, this may be due to more sedentary lifestyles, smoking, and excess weight caused by overeating; secondly, most of the published studies are carried out in populations in the United States (US) and Europe [14,15].

The glucose breath test (GBT), used to diagnose SIBO, is a good starting point that helps identify a group of patients who require specific treatment, usually antibiotics, before any carbohydrate intolerance may be diagnosed [16]. Bacterial overgrowth per se can cause secondary lactase deficiency and other malabsorption problems [17]; therefore, the presence of SIBO would invalidate fructose and lactose malabsorption tests [18,19]. For the present study, GBT was selected to assess SIBO among the patients given its less invasive nature [20], and to assure more reliable carbohydrate malabsorption results. It is important to consider that the origin of SIBO may be related to other underlying conditions in each patient [16,21], which should be analyzed in depth. Breath tests to assess the intestinal absorption of lactose and fructose are recommended when SIBO testing is negative or when symptoms persist after SIBO treatment. Breath tests that measure both hydrogen and methane have been used, as measuring methane values increases the sensitivity of this test [22]. Some authors state that around 20–30% of the population have positive methane levels when performing this type of test, and not measuring it could cause results to be misinterpreted [19]. The cut-off value for identifying fructose and lactose malabsorption is an increase above 20 ppm of H_2_ and/or a concentration greater than 12 ppm of CH_4_ before minute 90. If these values are observed without symptoms, fructose or lactose malabsorption is diagnosed after the ingestion of sugar, and if accompanied by symptoms, it is classified as intolerance [22].

In order to classify the patients, we also considered genetic variables that could influence the dietary treatment, as is the case of the lactose non-persistent (LNP) condition. LNP is present in a variable proportion worldwide, ranging from 5% in populations of Northern Europe to 100% in some Asian populations [9], and some authors affirm that around 75% of the world population present lactose malabsorption after age 30 [19]. Patients with LNP and lactose malabsorption were grouped together in order to provide a more specific nutritional treatment, given that lactose restriction may be necessary for symptom control [2,17]. In addition to identifying patients with secondary lactose malabsorption, recording the most frequent associated gastrointestinal (altered gut transit, abdominal distension, flatulence, abdominal pain) and extraintestinal symptoms (skin itching, articular pain, fatigue) is essential. Considering the high prevalence of LNP in our study population, genetic testing is relevant when prescribing long-term treatment and understanding how to guide patients in lactose reintroduction or not throughout their life. Identifying LNP cases is also important as lactose absorption is conditioned by the enzymatic capacity of each patient, and even after identifying and resolving other causes related to intestinal mucosal alterations, such as CD or SIBO, the level of malabsorption may depend on the genetic predisposition and absorptive capacity of each patient in the long term [23,24]. Ten out of eighteen patients of this study presented LNP genetics and also had lactose malabsorption. On the contrary, if a patient who does not carry LNP genes presents lactose malabsorption, other primary causes should be considered, such as CD [7], SIBO or parasitism [11]. In spite of the high prevalence of lactose malabsorption among the LNP group, some authors state that the correlation between the results of genetic testing and breath tests cannot be measured because each test measures different parameters—the genotypic expression of the lactase enzyme and the intestinal absorptive capacity per se [25]. Additionally, there are some limitations regarding the LNP genetic test. The A-22018 polymorphism has been described as protective in the European population when associated with the T-13910/A-22018 haplotype; however, it has been described as protective, independent of T-13910, in other populations. The A-22018 polymorphism has scientific evidence, but it is not as extensive as the other polymorphisms in the analysis. The test applied in this research does not evaluate congenital alactasia, nor secondary or transient hypolactasia [26]. However, a strength of this work is considering both genetic predisposition and intestinal malabsorption in order to design an adequate dietetic treatment for patients.

Genetic testing was also performed to determine mutations in the aldolase B (ALDOB) gene. Additionally, breath tests were used to measure the intestinal absorption of fructose. Hereditary fructose intolerance is a rare pathology usually identified in the early stages of life, in which, due to genetic mutations, the aldolase B enzyme is deficient, causing fructose accumulation in the intestine and other organs [27]. In the present study, only one patient was identified as a carrier of one copy (heterozygosity) for one of the seven main mutations of the study gene. Although this patient is a carrier of the mutation (which implies potential transmission to his offspring), it is very unlikely, in less than 1% of cases, that there might be another mutation that could cause the development of the disease [28]. Individuals in this condition are considered to have a normal fructose metabolism since an ALDOB activity level of approximately 50% is presumed to be sufficient for adequate function [29]. However, some authors state that these patients might exhibit elevated uric acid responses and potentially have mild defects in fructose metabolism, as well as an increased cardiometabolic risk, after ingesting moderate amounts of fructose, even in the absence of classical manifestations of the disease [29,30]. Based on the low prevalence of hereditary fructose intolerance and according to other authors, further genetic causes that could be related to the symptomatology and fructose malabsorption should also be considered, such as the presence of the HLA-DQ2/DQ8 haplotype, which is present in most individuals who have CD and is associated with gastrointestinal tract alterations, as mentioned below [8,11,24]. Hence, it is recommendable to focus the treatment for fructose malabsorption patients on the possible primary causes likely to be related to an alteration of the intestinal mucosa and responsible for the malabsorption of fructose, including celiac disease, parasitic infections, diet, SIBO, among others [11,24,31]. The HLA-DQ2/8 haplotype study is used to determine the genetic predisposition to developing CD [32] and a possible cause of gastrointestinal symptomatology [31]. Although confirmatory tests are required for the diagnosis of CD itself, with serology and duodenal biopsy being the gold standard [25], genetic testing is very reliable for ruling out CD and differentiating it from other pathologies such as a wheat allergy [32]. Currently, the recommended serological test for the diagnosis and monitoring of CD is the determination of anti-transglutaminase (ATGt) IgA antibodies, due to their high predictive value, high sensitivity, and specificity [3,33]. However, a disadvantage of this marker is that its results may vary depending on gluten consumption, and the number of people who eliminate or restrict certain foods from their diet, including gluten, is increasing, potentially resulting in false negatives [2,25].

According to some authors, CD does not necessarily have to develop for gastrointestinal symptoms related to gluten consumption to be present. It has been observed that patients who are HLA-DQ2/8-positive, when exposed to a gluten-containing diet, have accelerated intestinal transit and an altered mucosal barrier function compared to negative subjects [8,34]. Other authors indicate that when gluten was eliminated from the diet, HLA-DQ2/8-positive subjects showed greater improvement in depression and a lack of vitality compared to the HLA-DQ2/8-negative group, in whom a greater reduction of abdominal distension was observed [8]. Among the patients in this study, the presence of the HLA-DQ2/8 haplotype is a factor to be considered for the treatment, especially when other possible causes of symptoms, such as SIBO and poor diets based on ultra-processed foods, have been addressed without favorable results [31]. Differentiating gluten ingestion associated with GI symptoms from other pathologies with a similar symptomatology, such as intestinal bowel syndrome (IBS) or Crohn’s disease, is useful to provide a more accurate diagnosis and effective treatment [35]. Even in the absence of CD diagnosis, the presence of the HLA-DQ2/8 haplotype suggests that a gluten-free diet could improve the patients’ GI symptoms [36]. In agreement with other authors, when different treatments have failed and the patient’s symptoms remain, it is crucial to opt for a gluten-free diet as possible treatment, especially if limiting the ingestion of only one specific nutrient (in this case gluten) causes the patient’s quality of life and symptomatology improve a great deal [35,37].

A holistic and thorough assessment of the patients with GI pathologies must be carried out, considering the multifactorial aspects involved in the development of these pathologies. Genetic predispositions play a significant role, but so do environmental factors such as exposure to infections (*Giardia intestinalis* and *Helicobacter pylori*) and lifestyle choices (the level of stress and physical activity) [38,39]. Last but not least, the adoption of appropriate eating habits for each individual case is crucial, including in the prevention of recurrences, such as in the case of SIBO [40].

Genetic testing has proven to be a useful tool in identifying the possible therapeutical scenarios needed to design dietary management plans for patients with CGD, and it shows promising outcomes regarding unnecessary diet restriction and provides a shorter recovery time in specific cases. For individuals with persistent lactase, a progressive reintroduction of lactose is recommended once symptoms have improved. Conversely, in LNP patients, complete reintroduction is excluded, and permanent restrictions are necessary, as symptoms of intolerance are likely to recur with lactose consumption [24]. Additionally, genetic testing for HLA-DQ2/8 could be useful in determining if a GFD could ameliorate the patient’s symptoms after addressing other potential causes such as SIBO or intestinal infections. Especially in FM, a GFD should be considered as a possible answer to the secondary cause of malabsorption for patients with a positive result for HLA-DQ2/8. Several authors describe an improvement in gastrointestinal symptomatology among HLA DQ2/8-positive patients after adopting a gluten-free diet, even in the absence of a CD diagnosis [8,24].

## 5. Limitations of This Study

Despite the limitations of this study, including a sample size conditioned by the difficulties in recruitment and the great heterogeneity detected in terms of primary and secondary causes of CGD, which has made it difficult to define specific clinical patterns, this research provides a novel perspective regarding the use of genetic testing for the assessment and dietary management of CGD. The classification that has emerged from our genetic results combined with the breath tests is a starting point for guiding nutritional treatment based on identifying primary causes of malabsorption and considering them together with secondary causes and symptomatology, for a more complete approach to each case. The sample size limits the possibility of generalizing results; therefore, further research is needed. In this study, three genetic conditions have been considered as possible primary causes of malabsorption; however, we must not forget that there is a large array of other possibilities that could explain the origin of a patient’s symptoms (inflammatory bowel diseases, food allergies, unhealthy diets, anti-inflammatory treatments, parasites etc.).

## 6. Conclusions

As demonstrated by our genetics results, carbohydrate absorption and symptoms on admission, health professionals face very diverse scenarios in clinical practice, with situations in which primary and secondary causes are combined, which pose a challenge that requires orderly and sequential management to achieve the expected improvements in patients’ quality of life and diet. Stratification according to genetic causes of gastrointestinal disorders will guide the beginning of the intervention, and after that, assessing the rest of the possible causes will allow professionals to personalize and refine dietetic–nutritional management while avoiding unnecessary diet restrictions and reducing recovery times. Incorporating genetic analysis routinely into clinical management at an affordable price will make it possible to investigate new genetic associations with intermediate phenotypes and to establish different genetic profiles with different symptomatology and/or responses to treatment.

## Figures and Tables

**Table 1:** Sociodemographic and clinical characteristics of the patients.

Characteristics	Patients (*n* = 49)
*Gender*	
Female, *n* (%)	31 (63.3%)
Male, *n* (%)	18 (36.7%)
*Age*	
Years (mean)	41.9 years
Minimum—maximum	17–70 years
*Gastrointestinal symptoms*	
Diarrhea, *n* (%)	17 (34.7%)
Constipation, *n* (%)	10 (20.4%)
Mixed diarrhea and constipation, *n (%)*	14 (28.6%)
Altered gut transit, *n* (%)	41 (83.7%)
Abdominal distension ^1^, *n* (%)	39 (79.6%)
Flatulence ^1^, *n* (%)	34 (69.4%)
Abdominal pain ^1^, *n* (%)	34 (69.4%)
Borborygmi ^1^, *n* (%)	24 (49.0%)
Heavy digestion ^1^, *n* (%)	23 (46.9%)
Burping ^1^, *n* (%)	20 (40.8%)
Dyspepsia ^1^, *n* (%)	19 (38.8%)
Fullness ^1^, *n* (%)	17 (34.7%)
Extraintestinal symptoms	
Skin itching ^2^, *n* (%)	10 (20.4%)
Articular pain ^2^ *n* (%)	8 (16.3%)
Fatigue ^2^, *n* (%)	8 (16.3%)
Weight loss ^2^, *n* (%)	5 (10.2%)
Headache ^2^, *n* (%)	3 (6.1%)

^1^ Reported in this table are only those ≥ 5 on the visual analogue scale (VAS). ^2^ Reported as presence or absence of extraintestinal symptoms.

**Table 2:** Classification of phenotypes in relation to primary lactose-intolerance risk.

C/T-13910	G/A-22018	G/C-14010	G/C-13915	G/C-13907	*n* (%)	Phenotype
CT	GA	GG	TT	CC	25 (51.0%)	LP
TT	AA				6 (12.2%)	No risk
CC	GG	GG	TT	CC	18 (36.7%)	LNP Risk

LP: Lactase Persistence condition; LNP: Lactase Non-Persistence condition.

**Table 3:** Classification of celiac disease risk regarding HLA-DQ2/8 haplotypes.

Allele 1	Allele 2	*n* (%)	Level of Risk	*n* (%)
DQ2.5	DQ2.5	3 (6.3%)	High	6 (12.2%)
DQ2.5	DQ2.2	2 (4.1%)		
DQ2.2	DQ7	1 (2.0%)		
DQ2.5	DQ8	0 (0.0%)		
DQ2.2	DQ8	0 (0.0%)		
DQ8	DQ8	0 (0.0%)	Moderate	16 (32.7%)
DQ2.5	DQ7	0 (0.0%)		
DQ2.5	DQ-	2 (4.1%)		
DQ8	DQ7	1 (2.0%)		
DQ8	DQ-	1 (2.0%)		
DQ2half	-	12 (24.5%)		
DQ2.2	DQ2.2	0 (0.0%)	Low	2 (4.1%)
DQ2.2	DQ-	2 (4.1%)		
			TOTAL	24 (49.0%)

**Table 4:** Genetic test results for intestinal disorder markers and breath test results.

		Breath Test Positives		
Genetic Test Positives	*n*	LM *n* (%)	FM *n* (%)	SIBO * n (%)
LNP phenotype	18	10 (61.1%)	5 (27.8%)	6 (33.3%)
HLA of risk haplotypes	24	7 (29.2%)	11 (45.8%)	7 (29.2%)

LM: Lactose Malabsorption; FM: Fructose Malabsorption; SIBO: Small Intestine Bacterial Overgrowth. * With no results for malabsorption of lactose and fructose.

**Table 5:** Celiac disease risk classification and breath test results.

		Breath Test Positives		
CD Risk Level (HLA)	*n*	LM *n* (%)	FM *n* (%)	SIBO * n (%)
High	6	2 (33.3%)	2 (33.3%)	3 (50%)
Moderate	16	5 (31.3%)	8 (50%)	3 (18.8%)
Low	2	0 (%)	1 (50%)	1 (50%)

CD: Celiac Disease. * With no results for malabsorption of lactose and fructose.

## Data Availability

Restrictions apply to the availability of some or all data generated or analyzed during this study to preserve patient confidentiality or because they were used under license. The corresponding author will detail the restrictions and any conditions under which access to some data may be provided upon request.
